# A binational study of the association between white matter hyperintensities and functional outcome in stroke patients

**DOI:** 10.3389/fneur.2026.1712109

**Published:** 2026-03-17

**Authors:** Eva B. Aamodt, Martin Røvang, Mona K. Beyer, Karim Borei, Farhaan S. Vahidy, Thomas B. H. Potter

**Affiliations:** 1 Division of Radiology and Nuclear Medicine, Oslo University Hospital, Oslo, Norway; 2 Computational Radiology and Artificial Intelligence Unit, Oslo University Hospital, Oslo, Norway; 3 Department of Neurosurgery, Houston Methodist, Houston, TX, United States; 4 TIRR Memorial Hermann, Houston, TX, United States; 5 Center for Health Data Science and Analytics, Houston Methodist, Houston, TX, United States

**Keywords:** deep learning, dependency, MRI, stroke, white matter hyperintensities

## Abstract

**Background:**

Measures of white matter hyperintensities (WMHs) represent a crucial part of post-stroke outcome prediction. Automatic WMH segmentation has proven particularly challenging in stroke cases. Using an improved method for WMH segmentation that incorporates stroke lesions, we set out to explore factors associated with higher WMH burden, as well as the association between WMH burden and post-stroke dependency across two different countries that may demonstrate significant variation in radiological presentation.

**Methods:**

A total of 384 acute ischemic stroke (AIS) survivors from the Norwegian Cognitive Impairment After Stroke (Nor-COAST; NO) study and the Houston Methodist Registry of Neurological Endpoint Assessments among Patients with Ischemic and Hemorrhagic Stroke (REINAH; US) database were analyzed. MRI and clinical data were collected upon acute care hospital admission. WMHs were measured automatically using the nnU-Net methodology, taking into account the acute stroke lesion.

**Results:**

No significant difference in WMH percentage was found between sites. Factors associated with higher WMH burden included only age in NO, while in US, very high age (≥ 85), smoking, and being underweight were key factors. The two sites showed significant differences in demographics and clinical characteristics: the US cohort exhibited greater racial heterogeneity, higher body mass index (BMI) with more extremely obese patients, higher National Institutes of Health Stroke Scale (NIHSS) scores, and more thrombectomies, whereas the NO cohort exhibited more tobacco use, hypercholesterolemia, and longer stay at the hospital. Post-stroke dependency was initially associated with higher WMH percentage overall but only remained significant after adjusment in Norwegians aged ≥85, while in the US, dependency was driven by stroke severity and treatment after adjustment.

**Conclusion:**

Cohorts from the US and Norway exhibit no significant difference in WMH burden, but differ in the factors associated with WMHs.

## 1 Introduction

Despite significant advancements in stroke care leading to reduced mortality rates (1), acute ischemic stroke (AIS) remains a major source of long-term disability worldwide. Stroke survivors often face post-stroke dependency, requiring assistance for daily activities, with risk factors including advanced age, severity of stroke, living alone, cognitive impairment, physical inactivity, and recurrent stroke (2, 3). Understanding the factors that contribute to post-stroke dependency is essential for developing effective rehabilitation strategies and ensuring personalized stroke patient care.

White matter hyperintensities (WMHs) are common neuroimaging findings in AIS patients. WMHs are indicative of small vessel disease and are associated with age, hypertension, body-mass-index (BMI), diabetes, smoking, etc. (4, 5). While the exact mechanism behind how WMHs may influence post-stroke outcomes remains an area of active research, their presence is associated with cognitive decline (6–8), poor recovery, and increased dependency (9, 10). Automatic segmentation of WMHs from neuroimaging in AIS patients is particularly challenging due to their similar intensity profile to stroke lesions and other pathologies (11), highlighting the need for improved methods targeting this specific patient group.

Stroke outcomes have been found to differ across countries, with significant differences in both mortality and dependency following a stroke (12, 13). However, research on differences in radiological findings and their association with outcomes across ethno-racial groups is lacking. The current study utilizes data from both a Norwegian and a cohort from the United States (US) in order to examine stroke recovery across diverse healthcare systems, exploring universal and potential region-specific factors that influence post-stroke dependency.

This study aimed to explore factors associated with higher WMH burden, as well as the association between WMH burden and post-stroke dependency. We hypothesized that (i) factors linked to more WMH are the same in both Norway and the US, and (ii) a higher WMH burden would be associated with worse outcomes (greater dependency) after AIS in both Norway and the US.

## 2 Materials and methods

### 2.1 Sample and inclusion criteria

The current study is based on data from two datasets: the Norwegian Cognitive Impairment After Stroke study (Nor-COAST; NO) and the Houston Methodist Registry for Neurological Endpoint Assessment among Patients with Ischemic and Hemorrhagic Stroke (REINAH; US).

Nor-COAST is a prospective longitudinal multicenter cohort study that recruited patients hospitalized with acute stroke at five Norwegian stroke units. Patient recruitment started in May 2015 and was completed in March 2017. The study details are described elsewhere (41). Nor-COAST was approved by the regional committee for medical and health research, REK Nord (REK number: 2015/171), and registered on clinicaltrials.gov (NCT02650531). REK Nord has also approved this current sub-study (REK number: 2019/397). All participants provided written informed consent in accordance with the Declaration of Helsinki. If a participant was unable to give consent, written informed consent for participation was provided by a family proxy. Inclusion criteria were as follows: patients admitted with acute ischemic or hemorrhagic stroke within 1 week of symptom onset and diagnosed according to the World Health Organization (WHO) criteria or based on findings from computed tomography (CT) or magnetic resonance imaging (MRI); age over 18 years; fluent in a Scandinavian language; modified Rankin scale (mRS) < 5 before the stroke; and able and willing to perform an MRI. Exclusion criteria were as follows: not treated in the participating stroke units; symptoms explained by disorders other than ischemic brain infarcts or intracerebral hemorrhages; and expected survival of less than 3 months after stroke.

The compared cohort was obtained from Houston Methodist Registry of Neurological Endpoint Assessments among Patients with Ischemic and Hemorrhagic Stroke (REINAH), an observational registry of patients admitted to the Houston Methodist hospital system, which comprises seven certified stroke centers in and around Houston, Texas, USA. Drawing from electronic health records, the REINAH system automatically collects and cleans data from all adult patients (≥ 18 years) admitted with primary non-traumatic stroke, identified using encounter-based ICD-10 codes. The REINAH study was approved by the Houston Methodist Institutional Review Board (ID: PR00025034). Patient CT and MRI images collected during primary hospitalization are retained within REINAH, as are patient and encounter characteristics. Outcomes for patients with intracerebral hemorrhage are collected via phone at 30, 90, 180, and 365 days after discharge, with additional records for AIS patients provided by the Hospital Outcomes-based Prospective Endpoints in Stroke (HOPES), which collects 90-day functional outcomes using the mRS.

### 2.2 Clinical characteristics

In the NO cohort, study nurses and stroke physicians collected demographic and clinical data shortly after the acute stroke. Based on previous literature, the following baseline factors were included.

*Sociodemographic factors*: age, sex, ethnicity, years of education, tobacco use, and BMI. Age was categorized as ≤64, 65–74, 75–84, and ≥85 years.

*Comorbidities*: Hypercholesterolemia, hypertension, atrial fibrillation (AF), diabetes mellitus, and the Charlson comorbidity index (CCI). Hypercholesterolemia was defined as lipid-modifying treatment prior to admission and/or total cholesterol ≥ 6.2 mmol/L and/or LDL ≥ 4.1 mmol/L. Hypertension was defined as pre-stroke use of antihypertensive medication. AF was defined as a history of permanent or paroxysmal AF or atrial flutter recorded in medical records, and/or atrial flutter detected on electrocardiogram or telemetry during hospital stay. Diabetes mellitus was defined as a history of diabetes mellitus in medical records, pre-stroke use of antidiabetic medication, and/or HbA1c ≥ 6.5% at admission.

*Stroke characteristics*: stroke severity was assessed on day 1 after the acute stroke using the National Institute of Health Stroke Scale (NIHSS), length of stay at hospital (days), thrombolysis, and thrombectomy. NIHSS ranges from 0 to 42, with higher scores indicating more severe strokes. NIHSS was categorized as 0 = no stroke, 1–4 = minor, 5–15 = moderate, 16–20 = moderate to severe, and 21–42 = severe.

*Brain measures*: brain measures included WMH (cm^3^), intracranial volume (ICV, cm^3^), and WMH percentage (% of ICV). ICV was derived using FreeSurfer 6.0.1 (Martinos Center for Biomedical Imaging, Boston, MA, USA)(14).

In the US cohort, demographic and clinical data were collected by radiologists shortly after presentation with acute stroke, as part of standard care. Based on previous literature, the following baseline factors were included:

*Sociodemographic*: age, sex, ethnicity, state area deprivation index (ADI), tobacco use, and BMI. Age was categorized into ≤64, 65–74, 75–84, and ≥85.

*Comorbidities*: Hypercholesterolemia, hypertension, AF, diabetes mellitus, and CCI. Hypercholesterolemia was defined as lipid-modifying treatment prior to admission and/or if total cholesterol ≥ 6.2 and/or LDL ≥ 4.1. Hypertension was defined as pre-stroke use of antihypertensive medication. AF was defined as a history of permanent or paroxysmal AF or atrial flutter detected in electrocardiogram and described in medical records and/or permanent or paroxysmal AF or atrial flutter detected in electrocardiogram and/or telemetry during hospital stay. Diabetes mellitus was defined as a history of diabetes mellitus in medical records, pre-stroke use of antidiabetic medication, and/or HbA1c ≥ 6.5% at admission.

*Stroke characteristics*: stroke severity was measured on day 1 after the acute stroke using NIHSS, length of stay at hospital (days), thrombolysis, and thrombectomy. NIHSS was categorized as in the NO cohort.

*Brain measures*: the measures include WMH (cm^3^), ICV (cm^3^), and WMH percentage (% of ICV). ICV was derived using FSL SIENAX (University of Oxford, Oxford, UK) (15, 16).

### 2.3 Outcome assessment

Post-stroke dependency was measured using the mRS at 90 days post-stroke. mRS ranges from 0 to 6, with higher scores indicating worse disability (42). Dependency was considered as an mRS score of ≥3.

### 2.4 MRI acquisition

In the NO dataset, MRI was performed at baseline (within 2–7 days of the acute stroke). The assessments were done at five hospitals across the country (St. Olavs Hospital, Oslo University Hospital Ullevål, Vestre Viken Bærum Hospital, Haukeland Hospital, and Ålesund Hospital) using a single scanner at each site GE Discovery MR750, 3 T (GE Healthcare, Chicago, IL, USA); Siemens Biograph_mMR, 3 T (Siemens Healthineers, Erlangen, Germany); Philips Achieva dStream, 1.5 T (Philips Healthcare, Amsterdam, The Netherlands); Philips Achieva, 1.5 T (Philips Healthcare, Amsterdam, The Netherlands); Siemens Prisma, 3 T (Siemens Healthineers, Erlangen, Germany). The MRI protocol comprised 3D-T1 weighted, axial T2, 3D-Fluid Attenuated Inversion Recovery (FLAIR), diffusion-weighted imaging (DWI), and susceptibility-weighted imaging (SWI) sequences (details in Supplementary Table 1).

In the US dataset, clinical MRI images collected during the primary stroke encounter were obtained. If imaging was not available during the primary encounter, MRIs collected up to 365 days before admission or 30 days after discharge were considered. As REINAH collects system-wide imaging data from real-world admissions, no singular scanner make, model, or protocol was used. MRI images used for WMH assessment were required to include 3D T2-FLAIR images.

### 2.5 WMH segmentation

**Figure 1:**
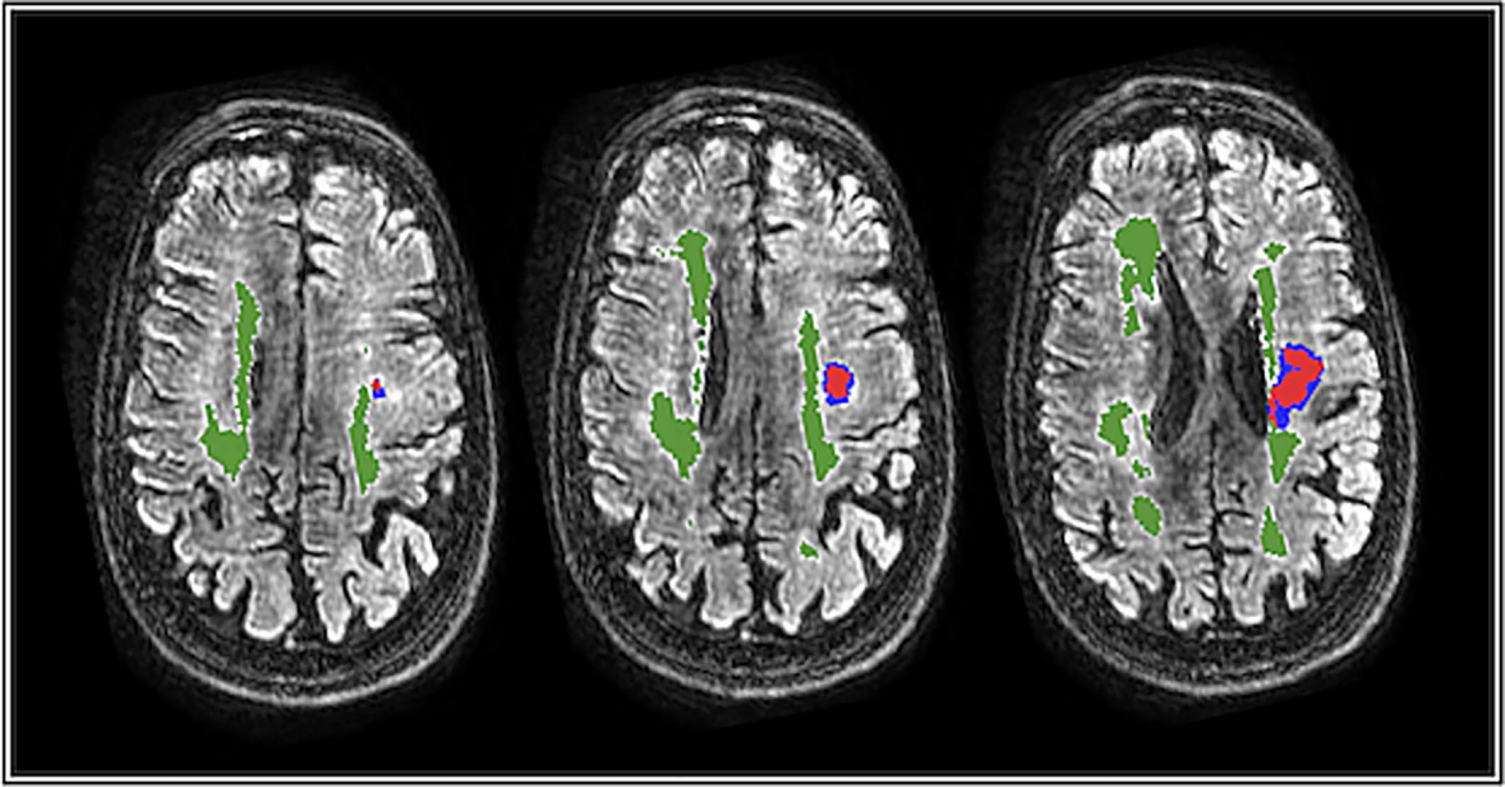
Example of WMH segmentation in green in a single patient across different horizontal cuts. Stroke lesion mask from DWI indicated in blue and red shows successful differentiation between WMH and stroke lesion.

WMH segmentation was performed using the tool “*Spotty”* [extended from Røvang et al. (17); Figure 1; Oslo University Hospital, Oslo, Norway], which uses nnU-Net methodology involving a self-configuring deep learning framework tailored for 3D volumetric medical image segmentation tasks, specifically applied to WMH segmentation. The preprocessing steps included converting MRI images from DICOM to NIfTI format, performing z-normalization of intensity values above zero, and resampling images to an isotropic resolution of 1 mm^3^ to standardize voxel dimensions. Data augmentation techniques, such as mirroring, zooming, Gaussian noise, Gaussian blur, brightness adjustments, and contrast changes, were applied to increase robustness.

The model employed a 3D U-Net-based architecture (Dynamic U-Net) implemented from the NVIDIA NGC catalog (NVIDIA Corporation, Santa Clara, CA, USA). The encoding feature map sizes were 64, 128, 256, 320, and 320, progressively capturing finer spatial details across layers. A combination of Dice Similarity Coefficient (DSC) loss and cross-entropy loss was used as the loss function. Training involved a patch-based approach with patch sizes of 128 × 128 × 128 and parameter optimization using the Adam optimizer. Automatic Mixed Precision (AMP) was utilized to enhance training speed and computational efficiency. Test-time augmentation (TTA) was employed during inference to improve segmentation robustness.

During inference, the model generated probability outputs that were thresholded at 0.5 to create binary segmentation masks. The segmentation performance was evaluated using metrics such as DSC, Hausdorff distance (HD95), average volume difference (AVD), recall, and F1-score. The nnU-Net demonstrated robust segmentation capabilities, achieving the highest DSC scores across both internal and external datasets. This result highlights the model’s ability to generalize effectively across different scanners and lesion sizes, particularly excelling in the segmentation of larger lesions. The methodology underscores the flexibility and effectiveness of nnU-Net in automating and standardizing medical imaging segmentation tasks.

The models were trained using a comprehensive dataset from the Norwegian Disease Dementia Initiative (DDI). This dataset comprised 441 participants, with a mean age of approximately 65 years, whose data were collected across five different sites and six scanners. The training process specifically utilized 3D FLAIR sequences, integral to assessing the performance of deep learning tools. Key models in the training dataset included the 3D nnU-Net, which demonstrated superior performance with an average DSC score of 0.76 ± 0.16 on internal validation data, and an internally developed 2.5D U-Net, which exhibited higher recall for small lesion detection, albeit with lower precision. Additionally, a Deep Bayesian Network, HyperMapp3r (Sunnybrook Research Institute, Toronoto, ON, Canada), was evaluated but showed limited performance, potentially due to a lack of retraining on this specific dataset. An external validation was performed using a smaller dataset from the Czech Republic.

To reduce false-positive segmentations and prevent misclassification of non-WMH pathology, including acute and subacute ischemic lesions with FLAIR hyperintensity, an iterative human-in-the-loop training procedure was used. The model was first applied to batches of 40 images, after which all segmentations were visually inspected and manually corrected by trained raters to remove incorrectly labelled regions. The corrected segmentations were then incorporated into subsequent model retraining. This process was repeated iteratively until the entire stroke dataset had been reviewed and incorporated. As a result, the final model output reflects WMH after explicit exclusion of visually identifiable ischemic lesions, despite reliance on single-modality FLAIR imaging.

### 2.6 Statistical analysis

Statistical analyses were performed using Python v3.11.11 (Python Software Foundation, Wilmington, DE, USA) and Stata v18.5 (StataCorp LLC, College Station, TX, USA). Means and standard deviations (SD) were calculated, and outliers and normality were checked using histograms. Simple comparisons between datasets were performed using Student’s *t*-tests for continuous variables; Mann–Whitney U test was used for skewed variables (NIHSS, WMH volume, WMH%, length of stay, and mRS), and *χ*^2^ tests for categorical variables; Fisher’s exact test was used when cell counts were small (ethnicity, BMI group, NIHSS group, and mortality at 90 days).

To test the first hypothesis, Student *t*-tests and chi-square analyses were first run between the NO and US sites. One multivariate (site, age, sex, ethnicity, smoking, BMI, hypercholesterolemia, hypertension, AF, diabetes mellitus, and CCI) linear regression model was fit for the whole dataset (NO plus US) to determine associations with the dependent variable of WMH percentage. The same linear regression (without site-variable) was then run per site (NO and US).

To test the second hypothesis, logistic regression models were fit for the primary outcome of mRS > =3 at 90 days: both univariate (WMH percentage) and multivariate (WMH percentage, site, age group, sex, ethnicity, smoking, hypercholesterolemia, NIHSS group, thrombolysis, and thrombectomy) models were run, and the models were run for the whole dataset (NO plus US) and per site (NO and US).

As a sensitivity analysis, effect modification by age and NIHSS was evaluated using multivariate logistic regression models including interaction terms between WMH percentage and age group or NIHSS group, respectively, rather than stratified models, in order to preserve statistical power and reduce overfitting. Dataset (NO or US) was included as a covariate in both models. To evaluate whether effect modification differed by dataset, an additional model including a three-way interaction between WMH percentage, age group, and dataset was also examined.

No formal correction for multiple hypothesis testing was used. The regression analyses were hypothesis-driven and based on two pre-specified outcomes, with models fit by site to assess consistency rather than independent hypothesis tests. Sensitivity analyses were conducted to evaluate the robustness of findings and were not intended for formal hypothesis testing.

## 3 Results

### 3.1 Comparisons of baseline characteristics and outcome between sites

**Figure 2:**
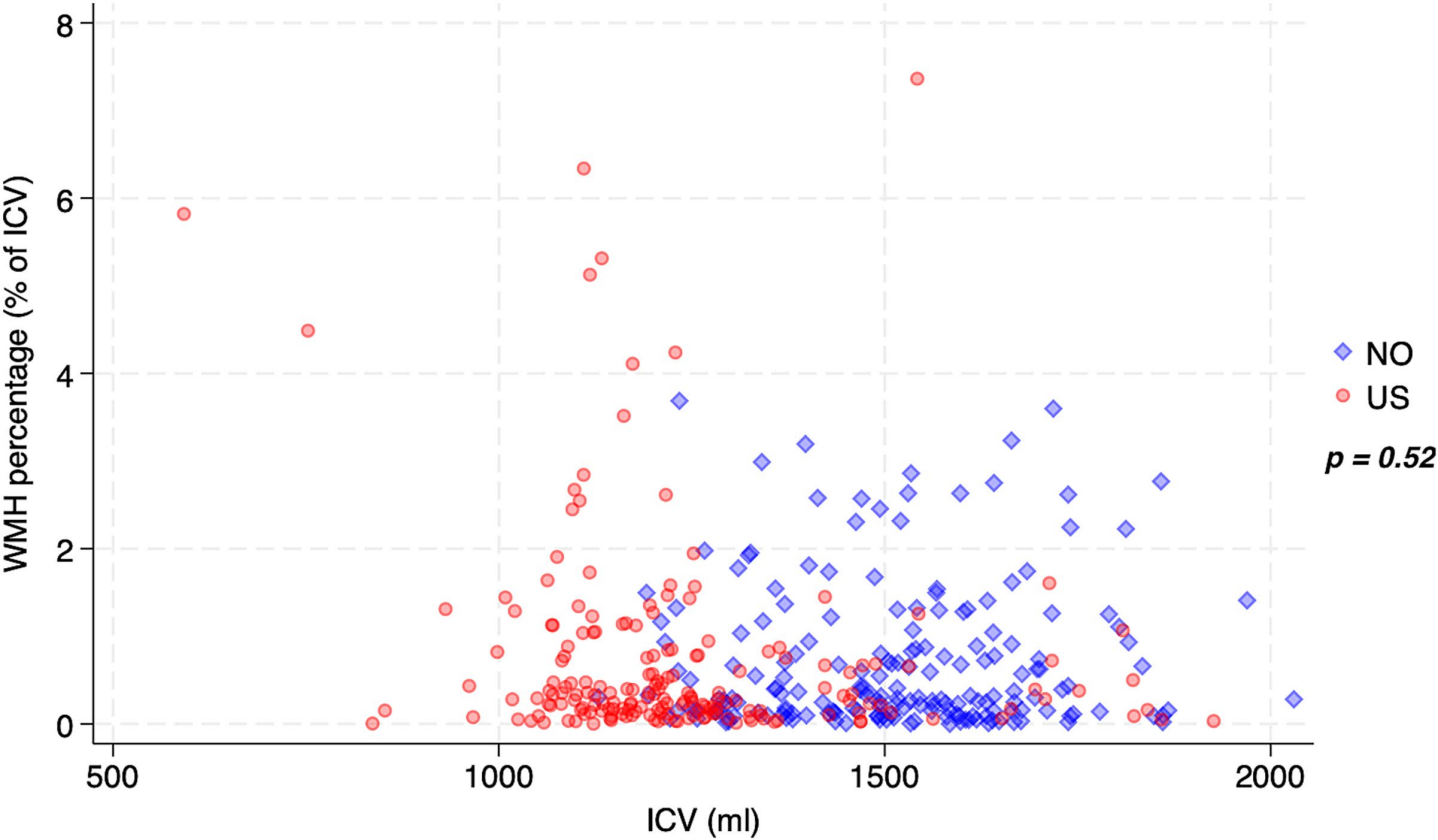
Plot showing WMH percentage over ICV (derived using different software) for both datasets. Non-significant *p*-value from Mann–Whitney U test of difference between WMH% across datasets. WMH, white matter hyperintensities; ICV, intracranial volume, mL, milliliter.

Table 1 summarizes demographic and clinical patient characteristics. A total of 384 patients were included, with 192 patients per site. Mean (SD) age was 71.44 (11.23) years, 172 (44.79%) were women, and the median (IQR) NIHSS score was 3 (1 to 6). Significant differences between the two sites were found for several factors; the US exhibited more racial heterogeneity (*p* < 0.001), higher numerical BMI (*p* < 0.001) with more extremely obese patients and fewer normal BMI patients (*p* = 0.002), higher NIHSS (*p* = 0.039), and more thrombectomies (*p* < 0.001). NO exhibited more tobacco use (*p* < 0.001), more cases of hypercholesterolemia (*p* < 0.001), longer stay at the hospital (*p* < 0.001), higher ICV volumes (*p* < 0.001) with a mean (SD) volume of 1512.58 (170.65) as compared to 1239.38 (201.05) in the US, and higher WMH volumes (*p* = 0.041) with a median (IQR) volume of 4.98 (1.81 to 15.45) as compared to 3.69 (1.65 to 10.23) in the US. No significant difference in WMH percentage was found between sites (*p* = 0.52; Figure 2), with a non-significantly higher median percentage of 0.32 (0.14 to 1.04) in NO compared to 0.31 (0.13 to 0.80) in the US.

**Table 1:** Baseline characteristics and outcomes.

	Full dataset	NO	US	*p*-value
N	384	192	192	
Sociodemographic				
Age*(years)	71.44 (11.23)	71.23 (10.89)	71.65 (11.23)	0.72
Age group*				0.95
0 to 64	91 (23.7%)	47 (24.48%)	44 (22.92%)	
65 to 74	140 (36.46%)	71 (36.98%)	69 (35.94%)	
75 to 84	107 (27.86%)	51 (26.56%)	56 (29.17%)	
85 +	46 (11.98%)	23 (11.98%)	23 (11.98%)	
Female*	172 (44.79%)	87 (45.31%)	85 (44.27%)	0.84
Ethnicity				**<0.001**
White	311 (80.99%)	190 (98.96%)	121 (63.02%)	
Black	53 (13.80%)	0 (0%)	53 (27.60%)	
Other	10 (2.60%)	2 (1.04%)	8 (4.17%)	
Asian	10 (2.60%)	0 (0%)	10 (5.21%)	
Education (years)	–	12.29 (3.66)	–	–
ADI (state)	–	–	4.39 (2.78)	–
Tobacco use	190 (49.61%)	118 (61.78%)	72 (27.50%)	**<0.001**
BMI	26.78 (5.12)	25.95 (4.39)	27.79 (5.75)	**<0.001**
BMI group				**0.002**
Underweight	13 (3.72%)	6 (3.12%)	7 (4.46%)	
Normal	124 (35.53%)	79 (41.15%)	45 (28.66%)	
Overweight	131 (37.54%)	73 (38.02%)	58 (36.94%)	
Obese	53 (15.19%)	28 (14.58%)	25 (15.92%)	
Extremely obese	28 (8.02%)	6 (3.12%)	22 (14.01%)	
Comorbidities				
Hypercholesterolemia	162 (42.19%)	98 (51.04%)	64 (33.33%)	**<0.001**
Hypertension*	193 (50.26%)	96 (50%)	97 (50.52%)	0.92
Atrial fibrillation*	82 (21.35%)	39 (20.31%)	43 (22.40%)	0.62
Diabetes mellitus*	67 (17.45%)	33 (17.19%)	34 (17.71%)	0.89
Charlson comorbidity	3.93 (2.33)	3.78 (1.72)	4.09 (2.81)	0.18
Stroke characteristics				
NIHSS (admission)	3 (1 to 6)	2 (1 to 5)	3 (1 to 7)	**0.039**
NIHSS group				**0.001**
No deficits	67 (17.54%)	38 (19.90%)	29 (15.18%)	
Minor	186 (48.69%)	101 (52.88%)	85 (44.50%)	
Moderate	101 (26.44%)	45 (23.56%)	56 (29.32%)	
Moderate to severe	16 (4.19%)	7 (3.66%)	9 (4.71%)	
Severe	12 (3.14%)	0 (0.00%)	12 (6.28%)	
Length of stay (days)	5 (3 to 7)	5.5 (4 to 8)	4 (2 to 6)	**<0.001**
Thrombolysis	110 (28.72%)	51 (26.70%)	59 (30.73%)	0.38
Thrombectomy	30 (7.81%)	5 (2.60%)	25 (13.02%)	**<0.001**
Brain measures				
Total grey matter vol. (cm^3^)		733.77 (52.43)	831.40 (325.70)	
Total white matter vol. (cm^3^)		451.38 (55.70)	663.70 (161.70)	
Total brain vol. (cm^3^)		1035.10 (79.48)	1495.20 (290.10)	
WMH vol. (cm^3^)	4.16 (1.78 to 12.10)	4.98 (1.81 to 15.45)	3.69 (1.65 to 19.23)	**0.041**
ICV (cm^3^)	1375.98 (231.06)	1512.58 (170.65)	1239.38 (201.05)	**<0.001**
WMH% (% of ICV)	0.32 (0.14 to 0.89)	0.32 (0.14 to 1.04)	0.31 (0.13 to 0.80)	0.52
Outcomes				
Dead at 90 days	5 (1.30%)	0 (0.00%)	5 (2.60%)	0.061
mRS at 90 days	2 (1 to 3)	2 (1 to 2)	2 (1 to 4)	0.052
mRS at 90 days-dependent	109 (28.39%)	34 (17.71%)	75 (39.06%)	**<0.001**

Continuous variables are presented as mean (SD) or median (IQR) for skewed distributions, and categorical variables as number (%). Comparisons between the NO and US datasets were performed using Student’s *t*-tests or Mann–Whitney U tests for continuous variables and *χ*^2^ or Fisher’s exact tests for categorical variables, as appropriate. No between-group comparisons were performed for total grey matter, white matter, or whole brain volumes due to site-specific processing pipelines. Comparisons were limited to measures directly related to the hypotheses. *, matched variables. *p*-values in bold, *p*<0.05 significance. mRS dependent, mRS ≥3; ADI, area deprivation index; BMI, body-mass-index; NIHSS, National Institutes of Health Stroke Scale; WMH, white matter hyperintensities; ICV, intracranial volume; mRS, modified Rankin scale.

A close-to-significant difference was found both for mortality at 90 days (*p* = 0.061), with 5 (100%) of deaths occurring in US as compared to zero in NO, and for mRS at 90 days (*p* = 0.052), with a median (IQR) mRS of 2 (1 to 2) in NO compared to a median (IQR) of 2 (1 to 4) in US. A significant difference between sites was found when mRS was dichotomized (*p* < 0.001), with significantly more dependent N (%) 75 (39.06%) in the US as compared to 34 (17.71%) in NO.

### 3.2 Hypothesis I: factors associated with WMH

Table 2 shows the results from the regression models. The multivariate linear regression for the full dataset revealed a positive association between WMH percentage and age group 65–74 [coef. 0.29, 95% CI (0.01 to 0.58), *p* = 0.045], 75–84 [coef. 0.65, 95% CI (0.34 to 0.95), *p* < 0.001], and ≥85 [coef. 1.15, 95% CI (0.77 to 1.53), *p* < 0.001], smoking [coef. 0.28, 95% CI (0.07 to 0.50), *p* = 0.011], and the BMI category of underweight [coef. 0.79, 95% CI (0.23 to 1.35), *p* = 0.006].

**Table 2:** Results from the regression models with the dependent variable of WMH percentage.

Multivariate regression—WMH percentage									
	Full dataset			NO			US		
	Coef.	95% CI	*p*-value	Coef.	95% CI	*p*-value	Coef.	95% CI	*p*-value
US (ref to no)	0.10	−0.14 to 0.34	0.41						
Age group (ref to 0 to 64)									
65 to 74	0.29	0.01 to 0.58	**0.045**	0.09	−0.25 to 0.43	0.60	0.39	−0.13 to 0.92	0.14
75 to 84	0.65	0.34 to 0.95	**<0.001**	0.54	0.17 to 0.90	**0.004**	0.53	−0.05 to 1.11	0.074
85 +	1.15	0.77 to 1.53	**<0.001**	0.76	0.31 to 1.21	**0.001**	1.42	0.74 to 2.11	**<0.001**
Female sex	0.08	−0.14 to 0.29	0.48	0.16	−0.07 to 0.39	0.18	−0.02	−0.40 to 0.36	0.92
Ethnicity (ref to white)									
Asian	0.38	−0.37 to 1.12	0.32	–	–	–	0.37	−0.53 to 1.26	0.42
Black	0.19	−0.75 to 1.12	0.70	–	–	–	0.31	−0.91 to 1.52	0.62
Other	0.26	−0.45 to 0.96	0.48	−0.01	−1.16 to 1.14	0.99	0.21	−0.67 to 1.08	0.64
Smoking status	0.28	0.07 to 0.50	**0.011**	0.06	−0.18 to 0.30	0.61	0.55	0.15 to 0.94	**0.007**
BMI group (ref to normal)									
Underweight	0.79	0.23 to 1.35	**0.006**	−0.10	−0.78 to 0.59	0.78	1.45	0.50 to 2.40	**0.003**
Overweight	−0.18	−0.42 to 0.06	0.14	−0.10	−0.35 to 0.16	0.46	−0.35	−0.79 to 0.09	0.12
Obese	0.06	−0.27 to 0.38	0.73	−0.20	−0.55 to 0.16	0.28	0.25	−0.34 to 0.84	0.41
Extremely obese	−0.23	−0.65 to 0.19	0.28	−0.30	−0.97 to 0.36	0.37	−0.29	−0.90 to 0.33	0.35
Hyperchol.	−0.10	−0.33 to 0.13	0.39	0.01	−0.23 to 0.25	0.93	−0.13	−0.59 to 0.32	0.57
Hypertension	0.01	−0.23 to 0.25	0.93	0.10	−0.15 to 0.35	0.43	−0.17	−0.63 to 0.29	0.47
AF	0.04	−0.22 to 0.30	0.78	0.02	−0.27 to 0.30	0.92	0.06	−0.41 to 0.54	0.80
Diabetes	0.08	−0.24 to 0.39	0.63	−0.04	−0.38 to 0.31	0.84	0.25	−0.34 to 0.84	0.41
Charlson CI	0.01	−0.05 to 0.05	0.94	0.08	−0.01 to 0.16	0.095	−0.02	−0.09 to 0.06	0.70

Results from multivariate linear regression models with the dependent variable of WMH percentage for the full dataset and the NO and US datasets. All variables shown were included simultaneously in the multivariable models. Significant p-values are in bold. WMH = white matter hyperintensities. Coef., coefficient; CI, confidence interval; Ref, reference to; BMI, body mass index; Hyperchol., hypercholesterolemia; AF, atrial fibrillation; Charlson CI., Charlson comorbidity index.

The multivariate model for NO alone showed changes from the full dataset, revealing a loss of significance for the age group 65 to 74, smoking, and the BMI category of underweight, leaving only age group 75–84 and 85 ≥ as significant factors.

The multivariate model for the US alone showed a change, revealing a loss of significance in the age groups 65–74 and 75–84.

### 3.3 Hypothesis II: the association of WMH with dependency for the full dataset

Univariate associations (Supplementary Table 2) for the whole dataset revealed that the odds of dependency increased with higher WMH percentage [OR 1.41, 95% CI (1.14 to 1.75), *p* = 0.002], although the effect weakened [OR 1.27, 95% CI (0.99 to 1.64), *p* = 0.064] in the multivariate model (Table 3). The multivariate model revealed significantly increased odds of poor functional outcome for the US site [OR 2.28, 95% CI (1.23 to 4.24), *p* = 0.009], age groups 75–84 years [OR 2.86, 95% CI (1.24 to 6.56), *p* = 0.013] and ≥85 years [OR 6.36, 95% CI (2.41 to 16.75), *p* < 0.001], NIHSS category of minor [OR 3.48, 95% CI (1.32 to 9.13), *p* = 0.011], moderate [OR 7.13, 95% CI (2.57 to 19.80), *p* < 0.001], moderate to severe [OR 17.06, 95% CI (3.74 to 77.87), *p* < 0.001], severe [OR 9.45, 95% CI (1.58 to 56.58), *p* = 0.014], and thrombectomy [OR 2.83, 95% CI (1.08 to 7.43), *p* = 0.035]. There was a significant decrease in odds with thrombolysis [OR 0.50, 95% CI (0.27 to 0.92), *p* = 0.026].

**Table 3:** Results from the multivariate regression models with the dependent variable of dependency.

Multivariate regression—dependency									
	Full dataset			NO			US		
	OR	95% CI	*p*-value	OR	95% CI	*p*-value	OR	95% CI	*p*-value
WMH %	1.27	0.99 to 1.64	0.064	1.51	0.91 to 2.50	0.11	1.18	0.88 to 1.59	0.28
US (ref to no)	2.28	1.23 to 4.24	**0.009**						
Age group (ref to 0 to 64)									
65 to 74	1.97	0.90 to 4.32	0.089	1.55	0.34 to 6.97	0.57	2.29	0.87 to 6.07	0.095
75 to 84	2.86	1.24 to 6.56	**0.013**	2.63	0.56 to 12.37	0.22	2.82	0.99 to 8.06	0.053
85 +	6.36	2.41 to 16.75	**<0.001**	14.05	2.78 to 71.03	**0.001**	2.39	0.65 to 8.78	0.19
Female sex	1.28	0.75 to 2.20	0.36	1.78	0.68 to 4.64	0.24	1.01	0.50 to 2.02	0.99
Ethnicity (ref to white)									
Asian	1.28	0.25 to 6.41	0.77	–	–	–	1.24	0.26 to 5.91	0.79
Black	1.10	0.14 to 8.94	0.93	–	–	–	1.21	0.14 to 10.68	0.86
Other	0.81	0.17 to 3.84	0.79	–	–	–	0.81	0.18 to 3.67	0.78
Smoking status	1.07	0.61 to 1.89	0.80	0.93	0.36 to 2.38	0.88	1.19	0.55 to 2.55	0.66
Hyperchol.	0.68	0.39 to 1.18	0.17	0.48	0.19 to 1.21	0.12	0.86	0.41 to 1.80	0.68
NIHSS (ref to 0)									
Minor	3.48	1.32 to 9.13	**0.011**	1.97	0.49 to 7.99	0.34	7.00	1.49 to 32.94	**0.014**
Moderate	7.13	2.57 to 19.80	**<0.001**	2.89	0.60 to 13.94	0.19	16.20	3.27 to 80.22	**0.001**
Moderate to severe	17.06	3.74 to 77.87	**<0.001**	9.10	0.98 to 84.34	0.052	43.66	4.21 to 452.90	**0.002**
Severe	9.45	1.58 to 56.58	**0.014**	-	-	-	27.92	3.35 to 232.63	**0.002**
Thrombolysis	0.50	0.27 to 0.92	**0.026**	0.52	0.16 to 1.71	0.28	0.44	0.20 to 0.96	**0.039**
Thrombectomy	2.83	1.08 to 7.43	**0.035**	0.03	0.24 to 29.91	0.43	2.42	0.80 to 7.33	0.12

Results from the multivariate logistic regression models with the dependent variable of dependency (mRS> = 3) for the full dataset and the NO and US datasets. All variables shown were included simultaneously in the multivariable models. Significant *p*-values are in bold. Subgroup-specific multivariable estimates are based on limited event counts and should be interpreted cautiously due to potential small-sample instability. WMH, white matter hyperintensities; OR, odds ratios; CI, confidence interval; Ref, reference to; Hyperchol., hypercholesterolemia; NIHSS, national institutes of health stroke scale.

### 3.4 Hypothesis II: the association of WMH with dependency between sites

Univariable associations for the NO cohort alone (Supplementary Table 2) were unchanged from the full dataset analysis, showing a significant increase in odds of dependency with WMH percentage [OR 2.03, 95% CI (1.36 to 3.02), *p* = 0.001]. However, adjustment for other variables (Table 3) resulted in a loss of significance for the 75 to 84 years age group, all NIHSS categories, thrombolysis, and thrombectomy, leaving only age group ≥85 as the significant factor for dependency.

Univariate associations for the US cohort alone were altered, leaving WMH percentage no longer a significant factor [OR 1.24, 95% CI (0.96 to 1.60), *p* = 0.11]. The multivariate model was also altered, revealing a loss of significance for all age groups and thrombectomy, leaving only NIHSS categories and thrombolysis as significant factors for dependency.

Stratified subgroup multivariable estimates demonstrated substantial variability with wide confidence intervals, reflecting limited event counts. Effect modification was therefore primarily evaluated using pooled interaction models. No evidence of effect modification by age group or NIHSS group was observed (all WMH% × age group and WMH% × NIHSS group interaction terms non-significant; global Wald tests non-significant; Supplementary Table 3), supporting the robustness of the primary findings. There was also no evidence of differential effect modification by dataset, as the three-way interaction between WMH burden, age group, and dataset was non-significant.

## 4 Discussion

This study aimed to use a stroke-specific WMH segmentation tool in order to explore factors associated with higher WMH percentage, as well as the association between WMH percentage and post-stroke dependency in a Norwegian and a US cohort. We found significant differences in demographics and clinical characteristics between sites, finding that the US cohort exhibited greater racial heterogeneity, higher BMI, higher NIHSS, and more thrombectomies, while the Norwegian cohort had more tobacco use, more hypercholesterolemia cases, longer stay at the hospital, and higher ICV and WMH volumes. There was no significant difference in the burden of WMH between sites, with similar percentages found. Factors associated with WMH burden included age, smoking, and being underweight overall, but site-specific analyses showed differing significance, with the Norwegian cohort showing association solely with age, and the US showing associations with age, smoking, and BMI. Higher WMH percentage was initially associated with post-stroke dependency across the dataset, but this significance weakened with multivariate adjustments, particularly in the US, where stroke severity and treatment were more impactful. In Norway, the association was maintained only for the age group ≥85, while in the US, WMH percentage ceased to be a significant factor, with NIHSS categories and thrombolysis remaining influential.

A significant difference in ICV volumes was found between the sites, but this is likely simply due to differences in the estimation methods used (18). When considering the percentage of the ICV affected by WMH, a difference between sites was also found; age, smoking, and being underweight were important factors in the US, but in Norway, only age was an important factor. High BMI has been consistently linked to increased WMH (19, 40); however, comparatively few have looked at the underweight. Regardless, patients in underweight BMI ranges fall in the same risk category as overweight or obese patients for cortical atrophy, which is correlated to higher WMH percentage (20). Further, WMH has shown to change in underweight patients with anorexia nervosa (21) and has shown a further dose-dependent relationship with smoking (22, 23). Associations have also been found with other lifestyle factors such as diet (24) and physical activity (25). Despite this, a large portion of contributing factors to WMH remain unexplained, with a recent study suggesting that 60% of the factors that shape WMH are unknown (26).

WMH has previously been shown to be associated with worse 3-month functional outcome (mRS) (27–29). We found that this association faded when accounting for other critical variables, including index stroke severity. This finding echoes previous studies that show a greater influence of WMH on patient outcome among patients with mild strokes and a reduced influence on outcome among patients with moderate to severe strokes (9). Our cohort, which presented with a mean admission NIHSS score of 4.55, includes mostly patients with moderate stroke, with a considerable proportion of moderate–severe or severe strokes. This may account for the diminished association between WMH and functional outcome when additional clinical and patient characteristics were accounted for.

Stratified subgroup multivariable estimates showed substantial variability with wide confidence intervals, reflecting limited event counts. Subgroup point estimates should therefore be interpreted cautiously. Effect modification was therefore primarily evaluated using pooled interaction-based sensitivity models; although higher-order interaction terms also exhibited wide confidence intervals due to sparse data, joint Wald tests did not demonstrate meaningful heterogeneity by age, NIHSS, or dataset. These findings support the robustness of the primary analyses while emphasizing the exploratory nature of the subgroup results.

The association between WMH and patient outcome may have disappeared in part due to higher age. WMH is highly associated with age and is a normal finding with healthy ageing, with a prevalence of 90% in ≥65 years of age (5). Age is also highly associated with functional outcome (30), and it seems that age, within the context of this project, is more tightly associated with patient outcome than WMH volumes. Advancing age is also associated with an increased risk of frailty, a clinical syndrome linked to greater dependency (31). A second factor that may have impacted the WMH-dependency associations is the administered treatment. Dependency was more frequent with thrombectomy. Thrombectomy is given to patients with strokes in larger arteries, meaning the stroke would affect more areas and lead to a higher risk of worse outcomes. The treatment is associated with a favorable outcome (32), although not for all patients; in a large study, only 37% of patients showed a good outcome (33). Conversely, dependency was less likely with thrombolysis, which matches previous reports (34). Considering the potential implications of treatment for stroke type and outcome, future models may seek to include additional measures of stroke severity, including infarct volume, affected vessel, and time-sensitive metrics like door-to-needle times.

Considering the two cohorts, hospitalization in the US, instead of Norway, was associated with dependency. In Norway, a 90-day mRS is usually 0–2 in around 73.5% and 3–5 in 26.5% (35). More dependency is typical in the US, with mRS of 0–2 in 61.8% and 3–5 in 38.2% (36). A study from Switzerland found that stroke patients in Switzerland were in inpatient settings on average 40 days longer than in the US, and that functioning was better at discharge (37). Length of stay in the hospital was on average 1 day longer in Norway in the current cohort, although this does not take into account subsequent rehabilitation duration or approaches.

In Norway, dependency was only associated with very high age (≥85), whereas in the US, dependency was not associated with age, but with stroke severity and treatment. Dependency is generally higher in the US, and stroke patients have a greater number of factors that may put them at risk for dependency. Norwegian patients, in contrast, show lower rates of dependence and only a single significant risk factor—older age. After the age of 85, it is very uncommon to stay independent, even if you have not suffered a stroke. One population-based study, not taking into account stroke, found that at age 85, 58% of men and 31% of women were independent, whereas at age 95, only 23% of men and 5% of women were still independent (38). As stroke severity and treatment are the only factors in the US, there seem to be more aspects at play. This could be down to more diversity in the US, for example, in ethnicity, socioeconomic status, access to rehabilitation, etc., factors associated with stroke severity, treatment, and post-stroke outcome. It is, however, surprising that age is not a significant factor in the US, as older age is a well established risk factor (36). This may, however, be due to the aforementioned impacts of stroke severity and the implications of treatment. It seems that in a cohort where most strokes are mild, age becomes a more important factor for dependency, while in a cohort where you have more diversity in stroke severity, the severity becomes a more important factor. Stroke severity has been found to be one of the most important predictive factors for post-stroke dependency in other cohorts (39).

### 4.1 Strengths and limitations

This study has several strengths and limitations. Conducting multinational studies like this one provides a broader understanding of disease patterns and associated risks by leveraging diverse populations and health systems. These studies enhance generalizability and allow for the examination of cross-cultural differences that may influence health outcomes. Additionally, multinational collaborations can drive innovations in treatment approaches and policymaking by integrating varied research methodologies and perspectives. Despite these strengths, a limitation worth noting is that different software packages were used across sites to derive intracranial volume (and brain measures). As segmentation algorithms and tissue definitions vary between software tools, this may limit the comparability of absolute volume estimates, although analyses within each site remain internally consistent. Future studies should aim for harmonized processing pipelines.

The newly developed segmentation software “*Spotty”* is a useful tool that works across countries and has been designed specifically for stroke patients. This study underscored the advantages of 3D nnU-Net in simplifying deployment through single-image input requirements, thereby facilitating automated large-scale WMH segmentation pertinent to clinical settings, especially in Alzheimer’s and small vessel disease research. Notably, limitations such as reduced performance on smaller lesions and data dependency on scanner type have previously been identified (17), highlighting areas for ongoing improvement and adaptation in real-world applications. Also, the US dataset comprised imaging data from real-world admissions, utilizing a diverse range of scanner makes and models. While this variability introduced a limitation by increasing the dataset’s variance, it simultaneously presented a significant strength. The results demonstrated that the *“Spotty”* tool was effective even in the presence of this variability, highlighting its robustness and practical applicability across heterogeneous imaging conditions. The reliance on single-modality FLAIR imaging in “*Spotty”* limits the differentiation of WMH from other pathologies that may also appear hyperintense on FLAIR. In this study, visual inspection and manual editing were used to refine the segmentations and iteratively inform the algorithm. Future studies should incorporate multimodal imaging, such as diffusion-weighted imaging for acute lesions, to support fully automated processing.

Another limitation worth mentioning is the exclusion of hemorrhagic strokes. Although they differ from ischemic strokes both in etiology and outcome, this still excludes a large portion of stroke patients.

The lack of frailty, education, and cognitive data is also worth mentioning, especially due to its strong association with post-stroke dependency. Future studies should include frailty, education, and cognitive testing, if possible.

## 5 Conclusion

The US and Norway exhibit differences in factors driving WMH burden, as well as factors associated with 90-day post-stroke dependency. WMH burden at AIS hospitalization is associated with post-stroke dependency.

## Data Availability

The raw data supporting the conclusions of this article will be made available by the authors upon reasonable request, subject to ethical approval and data protection regulations.
