# Supplementary material for: A binational study of the association between white matter hyperintensities and functional outcome in stroke patients
Source: Front Neurol. 2026 Mar 17;17:1712109. doi: 10.3389/fneur.2026.1712109 (PMC13035727; doi:10.3389/fneur.2026.1712109)
Supplement: Supplementary file 1 [file Table_1.docx]

Supplementary Material

**Supplementary table 1: List of MRI sequence parameters across hospitals in the NO dataset.**

| **Hospital** | **Sequence** | **Repetition**  **Time** | **Echo**  **Time** | **Inversion**  **Time** | **Flip**  **Angle** | **Rows** | **Columns** | **FOV** | **Slice**  **Thickness** |
| --- | --- | --- | --- | --- | --- | --- | --- | --- | --- |
| Oslo | T1_BRAVO_iso | 8.16 | 3.18 | 450 | 12 | 256 | 256 | 256x256 | 1 |
|  | CUBE_FLAIR | 8000 | 125.87 | 2092 | 90 | 256 | 256 | 256x256 | 1.2 |
|  | T2-PROPELLER | 6519 | 100.9 | NA | 142 | 512 | 512 | 512x512 | 4 |
|  | SWAN_3D | 37.2 | 23.24 | NA | 10 | 512 | 512 | 512x512 | 2 |
|  | DWI_TETRA | 3500 | 61.2 | NA | 90 | 256 | 256 | 256x256 | 6 |
| St. Olav | T1_MPRAGE_iso | 2300 | 2.01 | 900 | 9 | 256 | 256 | 256x256 | 1 |
|  | 3D_FLAIR | 5000 | 388 | 1800 | 120 | 256 | 256 | 256x256 | 1 |
|  | T2_TSE | 4200 | 81 | NA | 159 | 448 | 448 | 256x256 | 3 |
|  | SWI | 29 | 30 | NA | 15 | 512 | 384 | 512x384 | 2 |
|  | DWI | 6400 | 76 | NA | 90 | 128 | 128 | 128x128 | 4 |
| Haukeland | T1_MPRAGE_iso | 2300 | 2.03 | 900 | 9 | 256 | 256 | 256x256 | 1 |
|  | 3D_FLAIR | 5000 | 386 | 1800 | 120 | 256 | 256 | 256x256 | 1 |
|  | T2 | 4370 | 73 | NA | 150 | 448 | 448 | 448x448 | 3 |
|  | SWI | 29 | 20 | NA | 15 | 512 | 384 | 512x384 | 2 |
|  | DWI | 6400 | 76 | NA | 90 | 130 | 130 | 130x130 | 4 |
| Bærum | T1-3D | 7.47 | 3.44 | NA | 8 | 320 | 320 | 320x320 | 1.1 |
|  | 3D_FLAIR | 4800 | 284.1 | 1660 | 90 | 288 | 288 | 288x288 | 1.14 |
|  | T2 | 7069 | 100 | NA | 90 | 560 | 560 | 560x560 | 4 |
|  | SWI | 51 | 0 | NA | 20 | 672 | 672 | 672x672 | 2 |
|  | DWI | 4773 | 101 | NA | 90 | 224 | 224 | 224x224 | 4 |
| Ålesund | T1_3D | 25 | 4.602 | NA | 30 | 320 | 320 | 320x320 | 1 |
|  | 3D_FLAIR | 4800 | 250.5 | 1660 | 90 | 432 | 432 | 432x432 | 1.32 |
|  | T2 | 5463 | 100 | NA | 90 | 560 | 560 | 560x560 | 5 |
|  | VEN_BOLD | 24.56 | 34.7 | NA | 10 | 512 | 512 | 512x512 | 1 |
|  | DWI | 4623.9 | 108.3 | NA | 90 | 176 | 176 | 176x176 | 4 |

| **Supplementary table 2: Univariate regression models with the dependent variable of dependency** | | | |
| --- | --- | --- | --- |
| Variable: WMH% | OR | 95% CI | *p*-value |
| Full dataset | 1.41 | 1.14 to 1.75 | **0.002** |
| NO dataset | 2.03 | 1.36 to 3.02 | **0.001** |
| US dataset | 1.24 | 0.96 to 1.60 | 0.11 |
| Table: Results from the univariate logistic regression models with the dependent variable of dependency in the full dataset and the NO and US datasets. WMH = White matter hyperintensities. OR = odds ratios. CI = confidence interval. | | | |

**Supplementary table 3: Multivariate regression with the dependent variable of dependency – SENSITIVITY ANALYSES**

| Multivariate regression - Dependency – SENSITIVITY ANALYSES | | | | | | | | | |
| --- | --- | --- | --- | --- | --- | --- | --- | --- | --- |
| Model | 1: Age group # | | | 2: NIHSS group # | | | 3: Age group # NIHSS group # dataset | | |
|  | OR | 95% CI | *p*-value | OR | 95% CI | *p*-value | OR | 95% CI | *p*-value |
| WMH % | 1.04 | 0.29 to 3.71 | 0.95 | 0.72 | 0.27 to 1.94 | 0.52 | 0.20 | 0.01 to 109.48 | 0.61 |
| US (ref to NO) | 2.25 | 1.20 to 4.21 | **0.012** | 2.23 | 1.19 to 4.19 | **0.012** | 2.69 | 0.49 to 14.76 | 0.25 |
| US # WMH vol. (ref to NO). |  |  |  |  |  |  | 12.80 | 0.02 to 8800.01 | 0.44 |
| Age group (ref to 0 to 64) |  |  |  |  |  |  |  |  |  |
| 65 to 74 | 1.43 | 0.54 to 3.76 | 0.47 | 1.93 | 0.88 to 4.24 | 0.10 | 1.11 | 0.19 to 6.38 | 0.91 |
| 75 to 84 | 3.72 | 1.35 to 10.24 | **0.011** | 2.86 | 1.24 to 6.61 | **0.014** | 1.49 | 0.23 to 9.44 | 0.67 |
| 85 + | 5.36 | 1.58 to 18.19 | **0.007** | 6.50 | 2.45 to 17.25 | **<0.001** | 5.34 | 0.62 to 45.74 | 0.13 |
| Age group # WMH vol. (ref to 0 to 64) |  |  |  |  |  |  |  |  |  |
| 65 to 74 | 1.84 | 0.46 to 7.32 | 0.39 |  |  |  | 7.24 | 0.01 to 4325.90 | 0.54 |
| 75 to 84 | 0.91 | 0.24 to 3.41 | 0.89 |  |  |  | 7.51 | 0.01 to 4350.36 | 0.54 |
| 85 + | 1.34 | 0.34 to 5.25 | 0.68 |  |  |  | 12.71 | 0.02 to 7804.66 | 0.44 |
| Age group # US (ref to 0 to 64 and NO) |  |  |  |  |  |  |  |  |  |
| 65 to 74 |  |  |  |  |  |  | 1.66 | 0.21 to 13.16 | 0.63 |
| 75 to 84 |  |  |  |  |  |  | 2.32 | 0.27 to 19.97 | 0.44 |
| 85 + |  |  |  |  |  |  | 0.67 | 0.05 to 8.60 | 0.76 |
| Age group # US # WMH vol. (ref to 0 to 64 and NO) |  |  |  |  |  |  |  |  |  |
| 65 to 74 |  |  |  |  |  |  | 0.11 | 0.01 to 87.91 | 0.52 |
| 75 to 84 |  |  |  |  |  |  | 0.05 | 0.01 to 35.96 | 0.37 |
| 85 + |  |  |  |  |  |  | 0.03 | 0.01 to 24.75 | 0.31 |
| Female sex | 1.32 | 0.76 to 2.27 | 0.32 | 1.30 | 0.76 to 2.23 | 0.34 |  |  |  |
| Race (ref to white) |  |  |  |  |  |  |  |  |  |
| Asian | 1.37 | 0.27 to 7.05 | 0.70 | 1.25 | 0.24 to 6.38 | 0.79 |  |  |  |
| Black | 1.07 | 0.13 to 9.05 | 0.95 | 1.05 | 0.13 to 8.78 | 0.96 |  |  |  |
| Other | 0.80 | 0.17 to 3.90 | 0.79 | 0.78 | 0.16 to 3.78 | 0.76 |  |  |  |
| Smoking | 1.09 | 0.61 to 1.93 | 0.77 | 1.05 | 0.59 to 1.85 | 0.87 |  |  |  |
| Hyperchol. | 0.67 | 0.38 to 1.17 | 0.16 | 0.70 | 0.40 to 1.23 | 0.22 |  |  |  |
| NIHSS (ref to 0) |  |  |  |  |  |  |  |  |  |
| Minor | 3.40 | 1.27 to 9.08 | **0.015** | 1.99 | 0.65 to 6.11 | 0.23 |  |  |  |
| Moderate | 7.45 | 2.64 to 21.05 | **<0.001** | 4.93 | 1.49 to 16.30 | **0.009** |  |  |  |
| Moderate to severe | 17.02 | 3.69 to 78.52 | **<0.001** | 10.58 | 1.53 to 73.06 | **0.017** |  |  |  |
| Severe | 9.40 | 1.56 to 56.78 | **0.015** | 6.71 | 0.50 to 89.35 | 0.15 |  |  |  |
| NIHSS # WMH vol. (ref to 0) |  |  |  |  |  |  |  |  |  |
| Minor |  |  |  | 2.04 | 0.72 to 5.83 | 0.18 |  |  |  |
| Moderate |  |  |  | 1.64 | 0.53 to 5.04 | 0.39 |  |  |  |
| Moderate to severe |  |  |  | 1.98 | 0.24 to 16.56 | 0.53 |  |  |  |
| Severe |  |  |  | 1.60 | 0.08 to 30.43 | 0.76 |  |  |  |
| Thrombolysis | 0.49 | 0.26 to 0.91 | **0.024** | 0.50 | 0.27 to 0.94 | **0.031** |  |  |  |
| Thrombectomy | 2.90 | 1.10 to 7.68 | **0.031** | 2.80 | 1.06 to 7.39 | **0.038** |  |  |  |
| Wald test |  |  | 0.23 |  |  | 0.73 |  |  | 0.42 |
| Table 3: *Results from the sensitivity analyses of the multivariate logistic regression models with the dependent variable of dependency (mRS>=3). Model 1-2 adjusted for WMH percentage, age group, dataset, sex, race, smoking, hypercholesterolemia, NIHSS group, thrombolysis, and thrombectomy. Model-specific interaction terms are shown in the table. Significant p-values are in bold. Interaction term estimates are based on sparse data within some strata and should be interpreted cautiously; inference regarding effect modification is based on joint Wald tests. WMH = white matter hyperintensities. OR = odds ratios. CI = confidence interval. Ref = reference to. Hyperchol. = hypercholesterolemia. NIHSS = national institutes of health stroke scale.* | | | | | | | | | |

**
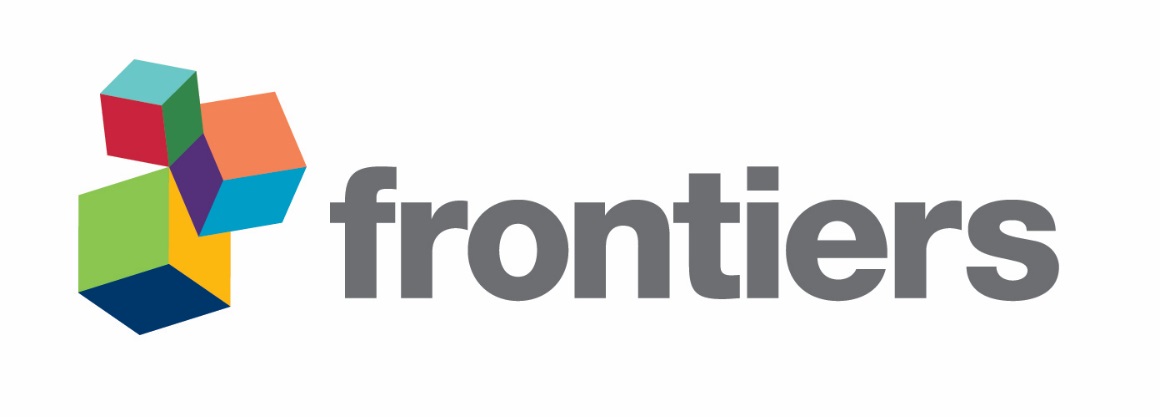
**
